# The Crosstalk between ROS and Autophagy in the Field of Transplantation Medicine

**DOI:** 10.1155/2017/7120962

**Published:** 2017-12-19

**Authors:** Anne C. Van Erp, Dane Hoeksma, Rolando A. Rebolledo, Petra J. Ottens, Ina Jochmans, Diethard Monbaliu, Jacques Pirenne, Henri G. D. Leuvenink, Jean-Paul Decuypere

**Affiliations:** ^1^Department of Surgery, University Medical Center Groningen, Groningen, Netherlands; ^2^Department of Digestive Surgery, Faculty of Medicine, Pontificia Universidad Católica de Chile, Santiago, Chile; ^3^Laboratory of Abdominal Transplantation, Department of Microbiology and Immunology, KU Leuven, Leuven, Belgium; ^4^Department of Abdominal Transplant Surgery, University Hospitals Leuven, Leuven, Belgium; ^5^Laboratory of Pediatrics, University Hospitals Leuven, Leuven, Belgium

## Abstract

Many factors during the transplantation process influence posttransplant graft function and survival, including donor type and age, graft preservation methods (cold storage, machine perfusion), and ischemia-reperfusion injury. Successively, they will lead to cellular and molecular alterations that determine cell and ultimately organ fate. Oxidative stress and autophagy are implicated in posttransplant outcome since they are both affected by the stress responses triggered in each step (donor, preservation, and recipient) of the transplantation process. Furthermore, oxidative stress influences autophagy and vice versa. Interestingly, both processes have positive as well as negative effects on graft outcome, suggesting they are tightly linked during the transplantation process. In this review, we discuss the importance, regulation and crosstalk of oxidative signals, and autophagy in the field of transplantation medicine.

## 1. Introduction

For patients with end-stage organ disease, organ transplantation has become the treatment of choice. However, the success of transplantation is limited by a global shortage of suitable organs as well as loss of grafts following transplantation due to primary nonfunction or rejection. The gap between supply and demand has steadily increased over the years and, as a result, so has the number of patients on the waiting list [1, 2]. This is an alarming increase, as organ transplantation significantly improves a patient's quality of life as well as survival rate when compared to patients who remain on the waiting list [3–5]. These problems can be addressed by increasing the use of older and higher risk donors, while simultaneously improving graft longevity.

Oxidative stress levels correlate with graft survival in all steps of the transplantation process including in the donor [6–16], during preservation [17, 18], and reperfusion in the recipient. In donation after brain death (DBD) donors, brain death pathophysiology leads to increased renal oxidative damage markers which correlate with acute rejection, delayed graft function (DGF), and allograft function [6–16]. In donation after cardiac death (DCD) donors, cardiac arrest causes warm ischemia which is associated with impaired graft function and higher mortality rates [6–8]. Increased oxidative stress markers are evident in organs from DCD donors, but no evidence is available correlating them with the outcome. Clinically proven donor treatments that benefit graft survival include dopamine administration and hypothermic cooling of DBD donors, of which the effects could be related to modulation of oxidative stress [19, 20].

Organ grafts suffer additional ischemic injury during preservation. Prolonged duration of cold ischemia is considered an independent risk factor for a nonfunctioning or dysfunctioning transplant, particularly in marginal or extended criteria donation (ECD) donors [21]. These side effects have recently led to the implementation of hypothermic machine perfusion (HMP). HMP has clear benefits over static cold storage, as evidenced by improved graft function and survival rates in kidney transplantation [22, 23], as well as reduced oxidative stress markers in experimental [24, 25] and clinical [26] liver preservation.

In the recipient, the mechanism of reperfusion injury, labeled ischemia-reperfusion (IR) injury (IRI), has been reported in most solid transplantable organs [27–31] and is mediated by reactive oxidative species (ROS) production most likely from donor-derived vascular cells [32, 33], followed by a second burst of ROS probably produced by the recipient's phagocytes [29, 34–40]. However, mitochondria are also implicated in ROS [29, 41–44] as well as nitric oxide (NO) production during IRI [45–47]. Despite overwhelming experimental evidence on the beneficial effects of attenuating ROS during this phase, clinical evidence remains limited.

Oxidative stress is a known inducer of autophagy. Even though autophagy regulation during the transplantation process is only starting to be understood, several autophagy modulators have already been implemented. In this review, we will first introduce autophagy in the context of the transplantation process and cover the current knowledge during each of these stages. Secondly, we will touch upon the complex, intertwined, and reciprocal relationship of oxidative stress and autophagy in the field of transplantation medicine while covering the therapeutic strategies that target each of these pathways.

## 2. Autophagy and Transplantation

### 2.1. Autophagy: Importance and Mechanisms

**Figure 1:**
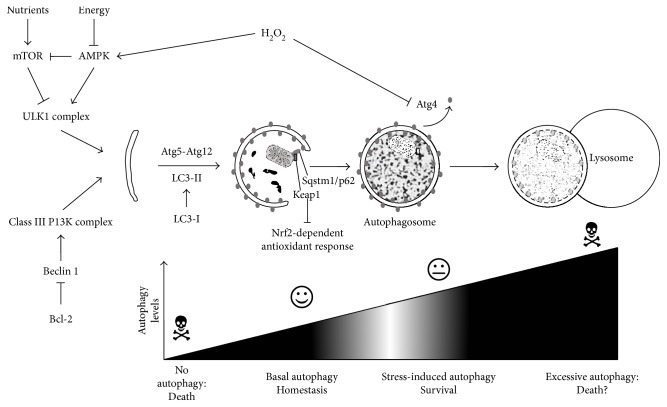
Overview of the autophagy process. Autophagy is initiated by the ULK1 complex, which is negatively regulated by mTOR, but positively by AMPK. This way it responds to nutrient or energy deprivation. In addition, class III PI3K complex requires Beclin 1, which is inhibited by Bcl-2. During elongation, Atg5-Atg12 and LC3-II are required. The latter is attached to the autophagosomal membranes. LC3-II will be delipidated on the outer membrane by Atg4 (a process inhibited by H_2_O_2_) but remains on the inner membrane and will be degraded inside the lysosomes. Mitochondria can be also degraded (mitophagy), via recruitment of Sqstm1/p62. The latter protein also recruits Keap1 for degradation, thereby enabling Nrf-2-dependent antioxidant transcription. Eventually, autophagosomes fuse with lysosomes, the cellular structures in which degradation takes place. The levels of autophagy determine the outcome on cellular injury and need to stay balanced in order not to provoke death.

Several pathways of autophagy exist [48], of which macroautophagy is the best studied (and which will simply be referred to as “autophagy” for the remainder of this manuscript). It involves the formation of double-membranous vesicular “autophagosomes” that occlude and transport the soon-to-be degraded material to the lysosomes (Figure 1). This process is normally constitutively active in cells, albeit at a low basal level, thereby maintaining cellular homeostasis (Figure 1). However, autophagy is stimulated upon stress through several signaling pathways, of which the mammalian target of rapamycin (mTOR) pathway is the most notable in response to nutrient stress. Energy deprivation attenuates mTOR signaling through the activation of AMP-activated kinase (AMPK), which also directly stimulates autophagy by the phosphorylation of the autophagy-initiating ULK1 complex. Another initiation complex is constituted by Vps34 (class III PI3K) and Beclin 1, an autophagy-specific BH3-only domain-containing protein that is inhibited by several antiapoptotic Bcl-2 family proteins (Figure 1). As such, apoptotic signals can also trigger autophagy, in which the autophagic response often precedes apoptosis as the first attempt to survival [49]. However, when autophagy fails, cells will eventually activate apoptosis, which may even occur with the help of the still active autophagic machinery. This shows that autophagy may switch from a prosurvival to a prodeath pathway under certain conditions [50], although the exact mechanism, context, and details on this autophagy-dependent cell death remain currently elusive.

Following initiation, the formation of autophagosomes is mediated by the Atg5-Atg12 complex and the formation of phosphatidylethanolamine-conjugated LC3 (“LC3-PE” or “LC3-II”) (Figure 1). The latter is generally used as a marker for autophagy, as it is distinguishable from its precursor LC3-I on Western blot and its fluorescent labeling allows visualization of autophagosomes as GFP-LC3 punctae. Despite delipidation of LC3-II on the outer membrane by Atg4, LC3-II remains attached to the inner autophagosomal membranes even after fusion of autophagosomes with lysosomes. Therefore, an increase of LC3-II or GFP-LC3 punctae can signify stimulation of autophagy or attenuation of the final steps in autophagy (e.g., inhibition of fusion), leading to an accumulation of autophagosomes without any true upregulation of autophagy. Therefore, prudency is advised when interpreting autophagy data because the dynamic character of the “autophagic flux” should be taken into consideration [51].

As the graft endures several types of stress during transplantation, it is evident that the protective properties of autophagy might be important in restoring cellular homeostasis and function in the organ grafts. Interestingly, DGF increases with donor (and recipient) age as aging leads to increased susceptibility towards cellular stress [52, 53]. An underlying mechanism for this increased vulnerability of aged organs is the age-related reduction in autophagy [52]. It has therefore been suggested that pharmacological stimulation of autophagy could reduce graft injury and promote function [54]. However, as excessive autophagy may detrimentally impact cellular fate through autophagy-dependent cell death (Figure 1), it is important to first understand the dynamics and role of autophagy to determine whether autophagy stimulation or inhibition is the best option in transplantation.

### 2.2. Autophagy in the Donor

**Figure 2:**
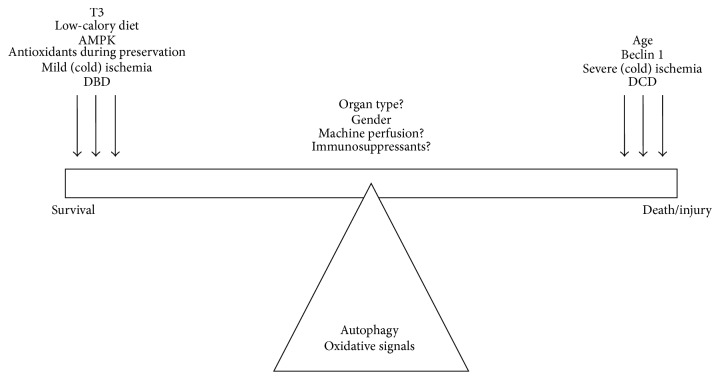
Regulation of oxidative signals and autophagy by transplantation-related factors. Excessive autophagy and/or oxidative stress can lead to increased graft injury and tilt the balance to the right. To tilt the balance towards survival, excessive signals need to be reduced towards protective levels of oxidative signals and autophagy.

The autophagic response in the donor is likely the result of donor-related characteristics including donor age, gender, comorbidities, and donor type of death. Of course, ischemic time is strongly prolonged in DCD compared to DBD grafts, which could partially explain the stronger injury in DCD grafts. This is important, since autophagy's dynamics and role (protective versus detrimental) during ischemic stress are likely dependent on the extent of ischemic injury, at least in the kidney [55, 56] and heart [57]. As DCD donors suffer more extensive anoxic injury compared to DBD donors, this could trigger autophagy-dependent cell death (Figure 2) and suggests that therapeutic strategies involving autophagy modulation are strongly donor type-dependent.

**Figure 3:**
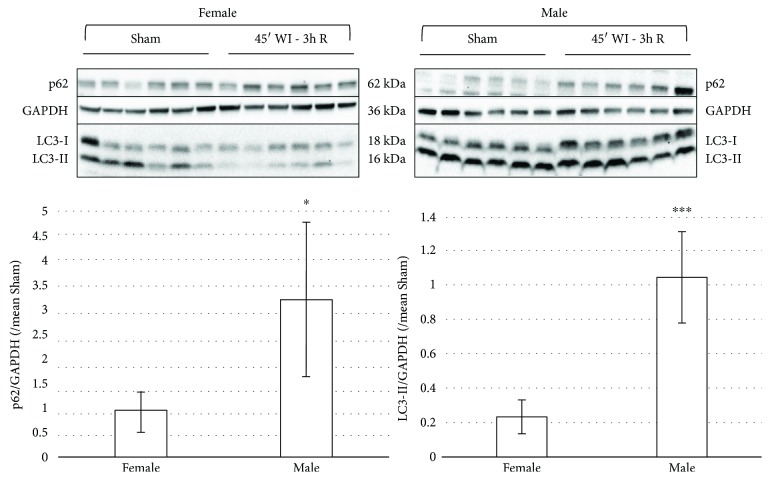
Gender differences in autophagy activation in response to ischemia-reperfusion injury. Western blot expression of autophagy-related proteins LC3-I, LC3-II, and Sqstm1/p62 in female and male Sprague-Dawley rat kidneys subjected to 45 min of warm ischemia (WI) followed by 3 h of reperfusion (3 h R). Each lane represents an independent experiment (*N* = 6). Quantification of p62 or LC3-II over GAPDH levels, compared to the mean of the corresponding Sham group. LC3-II and p62 are clearly lower in the WI group of females compared to the male WI group. Results are presented as mean ± SD (*N* = 6 per group) (^∗^*p* < 0.05; ^∗∗∗^*p* < 0.001).

Additionally, the higher posttransplant injury in ECD (e.g., older) kidneys may be attributed to a decline in autophagic activity with age [58–61], justifying autophagy stimulation as the preferred strategy in these donors. Compared to young mice, old mice showed a decreased autophagic response in terms of vacuole formation and elimination after stimulation of these processes with vinblastine and Triton X-100 [60]. In line with this, hypoxia-induced injury was reduced by starvation-induced autophagy in older kidneys [62]. Besides donor age, a less studied but seemingly equally important feature is the gender of the donor. In a cardiac IRI study in mice, males and females showed different autophagic activities: male mice show a decrease, and female mice an increase in LC3-II levels [61]. We have also observed gender differences in the kidney, with a decrease in male, but unchanged LC3-II levels in female Sprague-Dawley rats subjected to 45 min of ischemia followed by 3 h of reperfusion (Figure 3). As such, therapeutic strategies involving autophagy modulation may differ in organs coming from male and female donors (Figure 2).

### 2.3. Autophagy during Organ Preservation

Mixed reports exist on the effects of cold ischemia on autophagy. In mouse kidneys, cold ischemia resulted in increased autophagy markers [63]. Interestingly, repression of autophagic flux by means of lysosomal inhibitor bafilomycin A1 resulted in less apoptosis, suggesting that autophagy could trigger cell death during renal cold ischemia. Similar findings were observed in cold-preserved rat lungs [61], where prolonged preservation resulted in increased autophagy associated with cell death. Alternatively, decreased markers of autophagy were found following cold ischemia in marginal, steatotic rat livers [64–66]. Induction of autophagy in these marginal livers by means of melatonin and trimetazidine addition to the cold preservation medium improved organ quality as evidenced by lower levels of injury markers ALT and GLDH [66]. These benefits were attenuated when autophagy was suppressed with bafilomycin A1 [66]. Furthermore, oxygen insufflation of the cold preservation medium of marginal livers reversed the suppression of autophagy and functional impairment [65], suggesting beneficial effects of autophagy induction. These studies suggest that the differential effects of autophagy activation might be organ-dependent (Figure 2; see also Autophagy in the Recipient) and closely associated with organ function and cell death.

### 2.4. Autophagy in the Recipient

The role of autophagy in IRI is best studied in the context of the transplantation process. However, its dynamics and roles remain elusive and are likely dependent on different factors. Firstly, when studying the nature of autophagy dynamics, that is, whether autophagy is stimulated or attenuated during IRI, research suggests that autophagy is mostly upregulated in the heart [67] and the kidney [55], while findings in the liver are conflicting [64]. These discrepancies might be explained by several reasons (described in more detail in [55]), including the difficulty of measuring the dynamic process of autophagic flux in static samples. Indeed, an increase in LC3-II could equally well indicate stimulated autophagy as well as inhibited autophagic flux. *In vivo* experiments with chloroquine (an inhibitor of autophagosome-lysosome fusion, mimicking inhibited autophagic flux) suggest that autophagy inhibition might occur in the heart [68] and liver [69]. Moreover, as autophagy often fluctuates during prolonged stress, multiple time point postreperfusion should be investigated [55].

Secondly, conflicts arise when looking at the proposed role of autophagy during IRI. In hepatic IRI models, most studies indicate a protective role for autophagy [64], whereas both protective and detrimental roles are assigned to autophagy in the heart and kidney [55, 67]. Besides the difficulties in measuring autophagic flux, nonspecific chemical modulators of autophagy also have secondary effects on mTOR (e.g., rapamycin), PI3K (e.g., 3-methyladenine), or lysosomal and endocytic function (bafilomycin A1 and chloroquine) [55]. Even data in conditional autophagy knockout mice (e.g., atg5^−/−^) are important to interpret with caution. As autophagy is an important mechanism for basal cellular homeostasis in cells (Figure 1), any stress addition in these models will likely lead to more injury than in wild-type. In this context, proximal tubule-specific Atg5 KO mice displayed strange concentric membranous structures in targeted cells [70], suggesting unhealthy cells.

Thirdly, several reports propose a dual role for autophagy in IRI, dependent of the extent of the stress [55, 71]; that is, mild IR stress leads to protective autophagy stimulation, while severe stress could trigger a switch towards autophagy-dependent cell death (Figures 1 and 2). In this respect, the duration of ischemia prior to transplantation is an important factor to consider. The longer the ischemic period, the more severe the reperfusion injury, which seems associated mostly with a detrimental role for autophagy, at least in the kidneys [55]. Besides the extent of the stress, the type of stress that initiates autophagy is also important. This is evident in the heart, where autophagy-dependent cell death during reperfusion seems to be dependent on the levels of Beclin 1, in which initiation is likely determined by apoptotic factors. Protective autophagy on the other hand seems to be dependent on AMPK activation and would therefore be related to changes in energy status (Figure 2) [71].

Finally, autophagy during IRI is strongly determined by the degree of autophagy dependency of the organs and even of different cell types within an organ (Figure 2). In renal podocytes, for example, autophagy is much more important for cellular homeostasis (as these cells are postmitotic) than in tubular cells. Also, cardiomyocytes are strongly dependent on basal autophagy, while hepatocytes rely more on stress-induced autophagy. In this respect, liver cells might have different mechanisms controlling autophagy as they can tolerate more severe stress than other organs. This might explain the more consistent data regarding the protective role for autophagy in this organ compared to the kidney and the heart during IR.

## 3. Autophagy and Oxidative Stress in Transplantation

### 3.1. The Relationship between Autophagy and Oxidative Stress

Both oxidative stress and autophagy have been described as both protective and detrimental pathways in response to cellular stressors [34, 55]. Therefore, it is feasible that the fine balance of oxidative stress levels and autophagy activation plays an important role in the long-term function and survival of organ grafts. This makes modulation of these pathways interesting targets to predict or improve graft function and survival after transplantation.

Surprisingly, the only currently known direct redox-based regulation of autophagy is the inhibitory oxidation of Atg4 by H_2_O_2_, which suppresses the delipidation of LC3-II [72] (Figure 1). Furthermore, H_2_O_2_ is also proposed to stimulate autophagy initiation directly via regulation of AMPK [73] (Figure 1). In a slower, more indirect fashion, oxidative stress also regulates transcription of Beclin 1 and LC3 [54]. Together, these protective, proautophagic effects are achieved with subtle changes in ROS. Conversely, acute and persistent ROS production can oxidatively modify macromolecules in such a way that they are only partially degraded by the autophagic/lysosomal pathway. This produces an indestructible product known as lipofuscin, which accumulates within the lysosomes, hampers their function, and sustains or even exacerbates oxidative injury [74]. Together, this suggests that the amount of ROS produced will determine whether autophagy will be activated or inhibited and whether it prevents or amplifies further damage.

Besides the regulation of autophagy by oxidative molecules, autophagy reciprocally regulates oxidative signals. As it is a clearance mechanism, autophagy may remove oxidatively damaged macromolecules or even entire organelles. Upon excessive ROS production, mitochondria risk severe damage and need to be partially removed [75]. This occurs through the selective autophagic degradation of damaged mitochondrial fragments called “mitophagy” (Figure 1). This process involves the recruitment of PINK1 and Parkin to the outer mitochondrial membrane where they promote ubiquitination (Ub) of several mitochondrial proteins [76, 77]. This Ub signal serves as a recognition for Sqstm1/p62, which is an adapter protein for LC3-II (Figure 1). The link between ROS and mitophagy was confirmed in a study on heart failure in mice, describing that p53-induced impairment of mitophagy resulted in mitochondrial dysfunction and increased ROS production [76, 77].

In addition to mitophagy, autophagy is shown to regulate antioxidant responses through interactions of Sqstm1/p62 with Keap1. Sqstm1/p62 recruits Keap1 via interactions with ubiquitinated aggregates, after which Keap1 is degraded via autophagy. Degradation of Keap1 in turn prevents it from ubiquitinating and degrading Nrf2-dependent transcription factors, thereby enabling the antioxidant response [78] (Figure 1).

### 3.2. Autophagy and Oxidative Stress in the Donor

The accumulation of acute and chronic injuries in the donor leads to increased senescence and reduced organ quality. In ECD donors, this may be due to an increase in age-related oxidative damage (Figure 2) [16] as well as a reduction of autophagic activity and hence the inability to remove oxidatively damaged organelles. This link was shown in the kidneys of aged rats, where a high caloric diet exacerbated oxidative damage and aging, while autophagy was reduced [79]. The opposite was observed in the low caloric group [79]. The link between caloric intake and autophagy regulation is interesting, as it highlights how energy deprivation (i.e., glucose and amino acid stress) can initiate protection via autophagy-mediated pathways to restore cellular energy supplies (Figure 2) [73]. In response to starvation, increased activity of the mitochondrial apparatus in turn increases mitochondrial ROS production, which activates autophagy [73]. Interestingly, a proteomics study in brain-dead rodents indicated that both metabolic changes and mitochondrial dysfunction are the two major canonical pathways affected in the kidney of brain-dead donors [80]. Even though no direct line of evidence connects these alterations to autophagy, it is nonetheless feasible that autophagy is indeed modulated in DBD donors.

**Figure 4:**
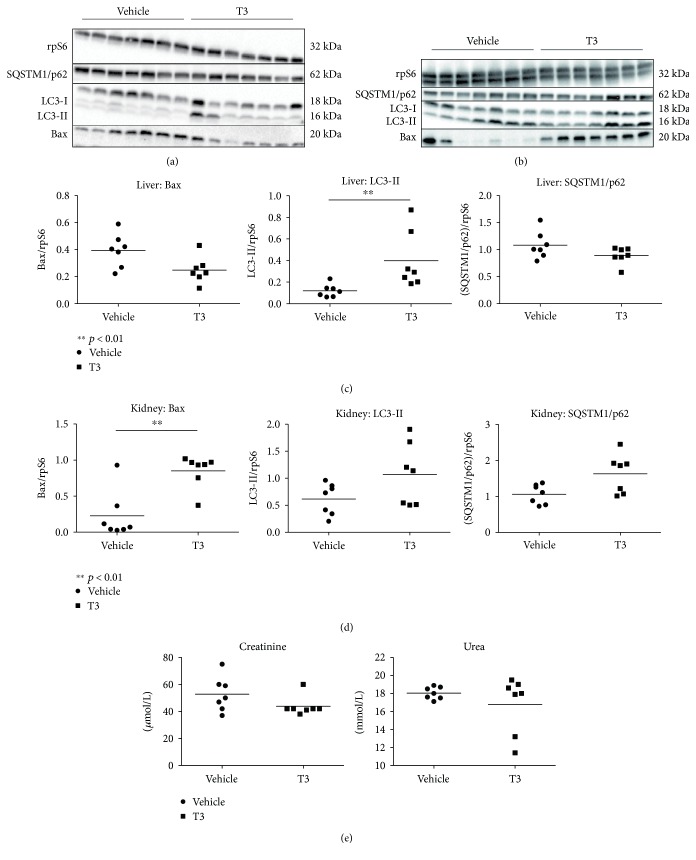
Posttranscriptional reduction of apoptosis and induction of autophagy in the liver of brain-dead rats following T_3_ treatment, yet no effects in the kidney. Western blot expression of proapoptotic protein Bax and autophagy-related proteins LC3-II and SQSTM1/p62 in the liver (a, c) and kidney (b, d) and renal injury markers in plasma (e) of T3- or vehicle-pretreated brain-dead rats. Each lane represents an independent experiment. Results are presented as mean ± SD (*N* = 7 per group) (^∗∗^*p* < 0.01).

**Figure 5:**
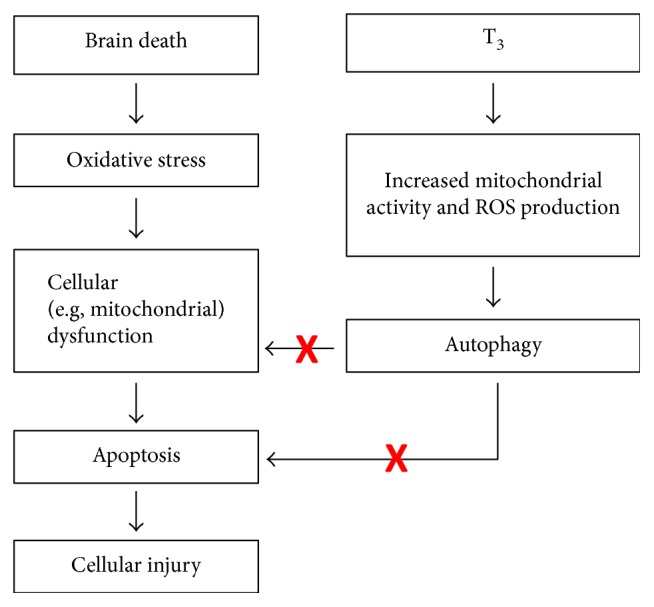
Proposed mechanism of T_3_ preconditioning during brain death in the liver.

A recent study from our group showed that the treatment of brain-dead rats with thyroid hormone T3, both a metabolic regulator and a powerful inducer of autophagy, improved liver function, while reducing apoptosis and oxidative stress [81]. In prosecution of this work [81], the protective effects of T3 preconditioning in brain-dead rats were indeed accompanied by an induction of autophagy in the liver, as evidenced by increased formation of LC3-II together with decreased (albeit nonsignificantly) levels of the autophagic substrate SQSTM1/p62 (Figure 4). Interestingly, in the kidney, T3 neither altered autophagy nor attenuated injury or apoptosis (Figure 4). Therefore, this suggests that T3-induced autophagy is not evident in the kidney and consequently, neither a reduction in apoptosis nor injury. In contrast, the reduction in apoptosis and injury markers in the liver does appear to be associated with increased hepatic autophagy, suggesting an important role for autophagy stimulation in the livers from brain-dead animals. We hypothesize that T3 boosts a transient peak in mitochondrial activity and ROS production, resulting in autophagy induction and subsequent protective effects during BD in the liver (Figure 5). This is supported by a recent study by Sinha et al. that showed that T3 induces autophagy via ROS-related pathways in the liver both *in vivo* and ex vivo [82, 83]. This phenomenon might not be evident in the brain-dead kidney due to extensive levels of oxidative stress [11], which might push the balance from a protective to a detrimental role for autophagy. This work therefore suggests that triggering oxidative pathways while simultaneously increasing autophagy could be an interesting strategy to improve liver function in DBD donors. In this respect, modulation of (mitochondrial) metabolism by compounds such as T3 might be the key to stimulation of transient ROS and subsequently protective autophagy activation (Figure 2).

### 3.3. Autophagy and Oxidative Stress during Preservation

Since most organs worldwide are still preserved on ice, several developments have been made to reduce ischemic injury during cold storage. The gold standard for preservation solutions in kidney, liver, pancreas and small bowel preservation is the University of Wisconsin (UW) storage solution [22, 84]. When comparing UW to HTK solution, the use of UW leads to decreased renal apoptosis and was linked to lower graft injury in human renal allografts [85]. UW contains, besides energy supplies (adenosine) and osmotic compounds, the antioxidants glutathione and allopurinol [84, 86] (Figure 2). However, the beneficial effects of these compounds are questionable considering their short life-spans and the fact that supplementation of glutathione to UW solution was unable to improve renal transplantation outcomes [84]. Nonetheless, a rodent study on kidney transplantation showed that treatment with hydrogen-rich saline (HRS), a novel antioxidant, immediately before ischemia attenuated transplantation-related renal injury and oxidative stress, while simultaneously inducing autophagic markers Beclin 1 and LC3-II [87]. Inhibition of autophagy with chloroquine attenuated these protective effects of HRS, suggesting that these effects were indeed autophagy-mediated and linked to oxidative stress [87]. Therefore, the addition of antioxidants to the preservation solution remains an interesting option to reduce graft injury (Figure 2). Conversely, prolonged cold ischemia of healthy kidneys in UW solution showed increased numbers of apoptotic and autophagic cells. In this case, the addition of bafilomycin A1 inhibited both autophagy and apoptosis [63]. These studies suggest that for the kidney the extent of cold ischemia injury could dictate whether either autophagy induction (in the case of shorter cold ischemia times) or inhibition (in the case of prolonged cold ischemia times) might be beneficial (Figure 2).

In the liver, cold storage of hepatic cells inhibited autophagic flux in both UW and Celsior solutions. However, only Celsior-stored cells reactivated autophagy upon reperfusion, while UW-stored cells displayed impaired lyso-autophagosomal fusion and elevated cell death [88]. Interestingly, pretreatment with simvastatin before cold ischemia led to the restoration of autophagy even in UW solution. This was accompanied by preserved cellular viability [88], suggesting possible benefits for hepatocytes when autophagy is induced during cold storage. These benefits might be linked to oxidative stress, as simvastatin-induced autophagy in cold-stored rat steatotic livers was accompanied by attenuation of oxidative stress [89], showing a link between ROS reduction and autophagy induction during cold liver preservation. In support of this, a study on liver transplantation in which the preservation medium (IGL-1) was enriched with trimetazidine, a fatty acid oxidation inhibitor, showed reduced oxidative stress yet activation of autophagy accompanied with reduced mitochondrial damage and hepatic injury [90]. These data therefore suggest that during cold storage of the liver, a strategy is preferred that simultaneously attenuates oxidative stress and stimulates autophagy.

The advantage of HMP over static cold storage has led to an increased implementation of this technique in the clinical setting. However, the lack of oxygen during HMP initiates high metabolic flux and subsequent high ROS production, particularly during the first minutes of reperfusion [91]. Therefore, benefits of oxygen addition to HMP solution are currently being investigated. Animal studies on liver and kidney HMP indicate that oxygenated HMP reduced mitochondrial flux and subsequent ROS production [91–93]. ROS production is further reduced when grafts are perfused at higher temperatures, such as subnormothermic (25°C) or normothermic (37°C) temperatures [94, 95]. Unfortunately, the effects of HMP on autophagy remain unexplored (Figure 2).

### 3.4. Autophagy and Oxidative Stress in the Recipient

As the extent of the oxidative response is dependent on ischemic duration, it is possible that the role of autophagy during IRI is dependent on the extent of oxidative stress. In this case, mild IR stress would trigger protective autophagy to counteract the oxidative damage experienced, for example, through autophagy-specific degradation of mitochondria (mitophagy). Regulation of mitophagy is thought to occur via oxidation of cardiolipin [29, 96–98], which plays a central role by mediating increased mitochondrial ROS release which serves as the signal for mitophagy initiation [99]. However, upon a very strong oxidative response (during severe reperfusion injury), an energy-dependent process as autophagy is not advised. In this case, autophagy stimulation could deprive much-needed energy, induce still elusive autophagy-dependent cell death mechanisms [100], or lead to the production of toxic lipofuscin (Figure 2) [74].

In the liver, IRI induced oxidative stress, autophagy, and apoptosis. These effects were attenuated by pretreatment with the antioxidant astaxanthin, potentially via modulation of the MAPK protein family [101]. In a study on renal IRI, increased oxidant production and autophagy markers were evident which suggests a tight and direct relationship between these two processes in which autophagy serves a protective role against renal oxidative damage [102]. In the heart, autophagic flux was analyzed during IRI in the presence of either H_2_O_2_ or the antioxidant N-2-mercaptopropionyl glycine (MPG). H_2_O_2_ increased, while MPG decreased autophagic flux, suggesting that autophagy is regulated by oxidative signals during cardiac IR. Interestingly, autophagy attenuation (by using heterozygous beclin 1^+/−^ mice) improved injury in this experimental setting [103]. Altogether, these findings imply that oxidative signals influence autophagy during IR and might be responsible for the exact role (protective or detrimental) autophagy plays in IRI, depending on the type of organ.

Several modulators of oxidative stress and autophagy have been clinically tested or are commonly used following transplantation. One method to combat the oxidative burst in recipients is ischemic postconditioning (IPoC), a technique that involves the temporal cessation of blood flow to a remote tissue such as a limb or locally via constriction of a nearby afferent artery. Despite promising preclinical results of local [104] and remote IPoC [105], clinical trials investigating both techniques did not report improved renal function after transplantation [104, 106]. Interestingly, IPoC also stimulates autophagy in the heart [107, 108], but this seems to depend on the postreperfusion time [109]. A clinical study on the addition of the antioxidant human recombinant SOD showed decreased rejection rates and improved survival following cadaveric kidney transplantation, despite mixed preclinical results [17, 110]. These results might be autophagy-dependent, as SOD overexpression resulted in attenuation of starvation-induced autophagy and apoptosis [111].

Many of the compounds given to transplant recipients as part of their immunosuppressive regimen also modulate autophagy. These include calcineurin inhibitors cyclosporine and tacrolimus, mTOR inhibitors sirolimus (rapamycin) and everolimus, mycophenolate mofetil (MMF), and corticosteroids [55, 112]. Cyclosporine is a known autophagy inducer that has now largely been replaced by tacrolimus as the treatment of choice in most European and American transplantation centers, mostly because of its proposed nephrotoxic side effects [112]. Interestingly, these side effects are thought to be related to increased autophagic clearance and autophagosome formation, phenomena possibly mediated via oxidative stress and apoptosis [92, 113]. Tacrolimus, on the other hand, was recently identified as an autophagy modulator that acts via activation of transcription factor EB, which in turn increases the expression of both autophagy and lysosomal genes [114, 115]. However, it is likely that this is associated with a mild, beneficial induction of oxidative stress [116]. The use of rapamycin as well as second generation mTOR inhibitors such as everolimus and deferolimus has been used as part of immunosuppressive therapy mostly for their ability to limit T-cell proliferation, but these compounds also influence autophagy by means of mTOR inhibition. However, the use of rapamycin in animal models of renal IRI and transplantation has yielded questionable results. Some studies suggest that rapamycin improves mitochondrial homeostasis and, subsequently, reduces ROS production and cellular senescence [46]. On the contrary, a study on renal transplant recipients suffering from DGF shows that patients who received rapamycin had significantly lower chance to resolve DGF [117]. Interestingly, combined treatment with tacrolimus or mycophenolate mofetil (MMP), an immunosuppressive drug that is known to activate chaperone-mediated autophagy, enhanced the positive effects of rapamycin [55, 118]. The mixed effect of rapamycin treatment might be attributed to the amount of injury and subsequent extent of autophagy activation [55]. Finally, corticosteroids have been part of most postoperative and maintenance immunosuppressive regimens over the past years [119]. Interestingly, methylprednisolone (MP) both suppresses and stimulates autophagy in animal models [55]. This difference could be related to the extent of the preceding injury, or could be dose- and time-dependent, as MP treatment has opposite effects on oxidative injury when it is administered acutely (beneficial) or chronically (damaging) in rat lungs [120]. If and how MP affects autophagy modulation posttransplantation and whether this is influenced by oxidative signals remains to be elucidated.

## 4. Conclusion

Oxidative stress is an important component of the transplantation process as well as a known inducer of autophagy. Although the exact mechanism behind the complex reciprocal relationship between oxidative stress and autophagy is only beginning to be understood, it seems to play a major role in the different roles autophagy and oxidative signals seem to play during transplantation. The potential protective properties of autophagy and low levels of oxidative stress, yet detrimental effects when excessive activation occurs (Figure 2), make these two processes interesting therapeutic or diagnostic targets in each step of the transplantation process, while their tight interaction supports the possibility to target both pathways with only one compound. However, how these processes are preferentially modulated depends on the specific step in the transplantation process, the type of organ, the age and gender of the donor, the ischemic time, and other contributing factors. Therefore, future research should try and decipher the complex, intertwined relationship of oxidative stress and autophagy during the transplantation process. Finding the optimal balance between autophagic and oxidative processes is crucial for the optimization of cellular longevity and thereby graft survival.
